# Performance of re-used pacemakers and implantable cardioverter defibrillators compared with new devices at Groote Schuur Hospital in Cape Town, South Africa

**DOI:** 10.5830/CVJA-2015-048

**Published:** 2015

**Authors:** Zimasa V Jama, Ashley Chin, Bongani M Mayosi, Motasim Badri

**Affiliations:** The Cardiac Clinic, Department of Medicine, Groote Schuur Hospital, Cape Town, South Africa; The Cardiac Clinic, Department of Medicine, Groote Schuur Hospital, Cape Town, South Africa; The Cardiac Clinic, Department of Medicine, Groote Schuur Hospital, Cape Town, South Africa; College of Medicine, King Saudi Bin, Abdulaziz University for Medical Sciences, Riyadh, Kingdom of Saudi Arabia

**Keywords:** re-used devices, pacemakers, ICDs, performance, safety

## Abstract

**Objectives:**

Little is known about the performance of re-used pacemakers and implantable cardioverter defibrillators (ICDs) in Africa. We sought to compare the risk of infection and the rate of malfunction of re-used pacemakers and ICDs with new devices implanted at Groote Schuur Hospital in Cape Town, South Africa.

**Methods:**

This was a retrospective case comparison study of the performance of re-used pacemakers and ICDs in comparison with new devices implanted at Groote Schuur Hospital over a 10-year period. The outcomes were incidence of device infection, device malfunction, early battery depletion, and device removal due to infection, malfunction, or early battery depletion.

**Results:**

Data for 126 devices implanted in 126 patients between 2003 and 2013 were analysed, of which 102 (81%) were pacemakers (51 re-used and 51 new) and 24 (19%) were ICDs (12 re-used and 12 new). There was no device infection, malfunction, early battery depletion or device removal in either the re-used or new pacemaker groups over the median follow up of 15.1 months [interquartile range (IQR), 1.3–36.24 months] for the re-used pacemakers, and 55.8 months (IQR, 20.3–77.8 months) for the new pacemakers. In the ICD group, no device infection occurred over a median follow up of 35.9 months (IQR, 17.0–70.9 months) for the re-used ICDs and 45.7 months (IQR, 37.6–53.7 months) for the new ICDs. One device delivered inappropriate shocks, which resolved without intervention and with no harm to the patient. This re-used ICD subsequently needed generator replacement 14 months later. In both the pacemaker and ICD groups, there were no procedure-non-related infections documented for the respective follow-up periods.

**Conclusion:**

No significant differences were found in performance between re-used and new pacemakers and ICDs with regard to infection rates, device malfunction, battery life and device removal for complications. Pacemaker and ICD re-use is feasible and safe and is a viable option for patients with bradyarrhythmias and tachyarrthythmias.

## Objectives

Pacemaker implantation is an effective tool to treat bradyarrhythmias, and implantable cardioverter defibrillators (ICD) reduce mortality in patients at high risk of sudden death.^1^ The challenge with pacemakers and ICDs is the high cost of these devices. The pacemaker generator, in its most basic form, costs US$2 500–3 000 and leads cost US$800–1 000.^2^ An ICD generator costs US$20 000–40 000 and leads cost over US$10 000.2 The high cost of pacemakers and ICDs has resulted in limited access of deserving patients in poor countries to these life-saving interventions.^3^-^5^

Mond *et al.*^6^ demonstrated an increase in pacemaker and ICD implantation rates in all countries that participated in the World Survey of Cardiac Pacing in 2009. Despite this increase in implantation rates, there was a huge difference in the number of implants between the developed and underprivileged countries, with more implants in the developed world.^6^ This disparity was explained mainly by the high cost of these devices.^6^

Re-use of cardiac pacemakers has been practiced since the early 1970s.^7^ The major concern with this practice is the risk of device infection and malfunction.^8^-^11^ Device infection is the most feared complication of cardiac device re-use and is thought to be associated with case fatality rates between 2.6 and 18%.^12^-^14^ However, some studies from America, Europe and Asia that examined the performance of re-used pacemakers and ICDs have shown no significant difference in infection or mortality rates between patients who received re-used and new devices.^14^-^22^

The aim of this study was to investigate the performance of re-used pacemakers and ICDs at Groote Schuur Hospital, Cape Town, South Africa.

## Methods

**Figure 1.**
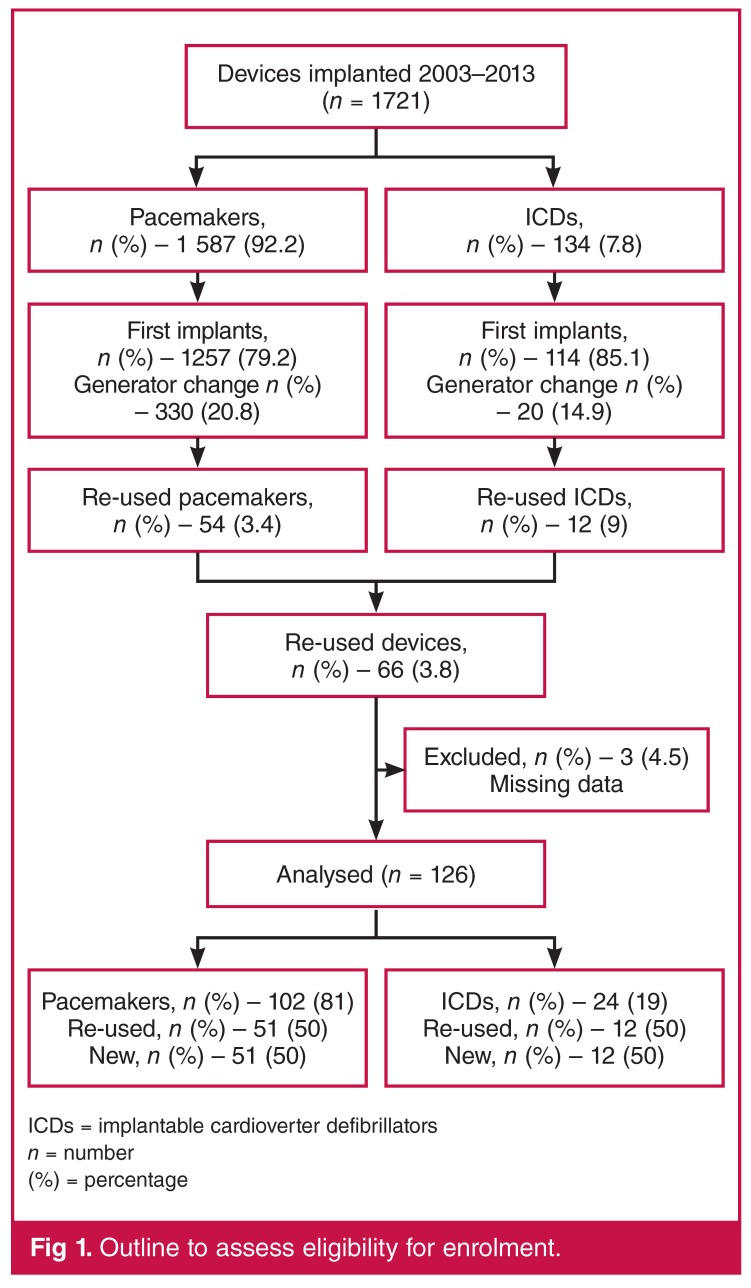
Outline to assess eligibility for enrolment.

This was a retrospective case comparison study of performance of re-used versus new pacemakers and ICDs at Groote Schuur Hospital, Cape Town, South Africa. We included consecutive devices that were implanted between 1 January 2003 and 1 January 2013. As shown in (Fig. 1, there were 1 721 devices implanted during that time, of which 1 587 (92.2%) were pacemakers and 134 (7.8%) were ICDs. Of the 1 587 pacemakers, 1 257 (79.2%) were new implants and 330 (20.8%) were generator replacements. Of the 134 ICDs, 114 (85.1%) were new implants and 20 (14.9%) were generator replacements.

There were 54 (3.4%) re-used pacemakers and 12 (9%) re-used ICDs implanted during this period, with a total number of 66 (3.8%) re-used devices implanted, as shown in Fig. 1. Patients with re-used devices (cases) were then matched by age, gender and date of implantation on a 1:1 basis to patients with new devices (controls). In the pacemaker group, cases and controls were matched to the same month of implantation, and for the ICD group, to the same year of implantation.

Devices for re-use were obtained from cadaveric donors. They were inspected for external damage and tested for remaining battery life. Devices with less than two years of battery life remaining and/ or with external evidence of damage were not re-used. Only devices with two or more years of battery life remaining with no evidence of external damage were eligible for re-use.

The eligible devices were sterilised by immersion in biozyme for 24 hours, followed by peroxide for a further 24 hours and then orthozyme for another 24 hours. After the three days of chemical treatment, the devices were dried out using pressurised air and subsequently subjected to gas sterilisation. In the gas sterilisation unit, they were put in a machine with ethylene oxide for 4.5 hours and irradiated for two cycles of 30 minutes, three days apart.

After device sterilisation, all devices were checked by a cardiac technologist in the department for any visual defects and for device longevity, and were tested to determine whether they were functioning appropriately for re-use. Device manufacturer’s personnel were not involved in this process.

A cardiac technologist or cardiology register in training was present at every implant procedure. Standard measurements were obtained during the implant after lead positioning (capture thresholds, battery life, sensitivities and lead impedances) and again prior to discharge.

Re-used pacemakers were implanted mainly in elderly patients with multiple co-morbidities such as advanced cancer (on treatment or in remission), cerebrovascular accident (CVA), advanced chronic obstructive pulmonary disease (COPD), dementia and/or a poor baseline level of functioning (mostly bed bound) who were expected to have a significantly reduced life expectancy. Re-used ICDs were implanted in patients who met the secondary prevention criteria for sudden death, and co-morbidity was not a factor in determining who received a re-used ICD.

The inherent difference between patients who received re-used pacemakers compared to those who had new pacemakers led us not to compare the outcome of patients in the two groups. The units of analysis were the devices themselves. Every patient provided a written informed consent for implantation of the device.

The devices were implanted by a cardiac electrophysiologist, cardiologist or a cardiology senior registrar. Prior to implantation, patients received 1 g of intravenous infusion of cefazolin as prophylaxis. Patients were discharged from hospital the following day provided there were no complications and were followed up in the pacemaker clinic at three months and yearly thereafter. Patients with ICDs were followed up more frequently at three- to four-monthly intervals.

## Outcomes

The outcomes of interest were procedure-related infection, device malfunction, early battery depletion, and device explantation for infection, malfunction and/or battery depletion. The definitions of the outcomes are as follows.

Procedure-related infection: infections were classified into four types:^23^ (1) right-sided endocarditis with lead involvement; (2) sepsis with evidence of involvement of the lead and implantation pocket; (3) involvement of the pacemaker implantation pocket; and (4) involvement of the lead or generator. Infections were considered early if the onset of illness was within the first month of implantation, and late if the onset of illness was after the first month to a year after implantation.^23^ Infections that occurred after a year of implantation were considered not to be related to the procedure.^23^
Device malfunction was defined as failure of the device to accomplish the desired role, e.g. in the case of an ICD, not able to sense ventricular tachycardia/fibrillation and deliver appropriate treatment. In the case of a pacemaker, device malfunction was defined as inability to sense or pace when required.
Early battery depletion was defined as battery depletion within six years of implantation for new devices. For re-used devices, early battery depletion was defined as battery depletion within one to two years of implantation for those with two to four years of battery life remaining, and within two years of implantation for those with four years or more of battery life remaining at the time of implantation, provided this depletion was not explained by high pacing outputs or abnormal electrode impedance.
Device explantation for infection, malfunction and/or battery depletion involved removal of the pacemaker or ICD due to infection, malfunction or early battery depletion.

## Data extraction

The cardiac clinic electrophysiology database was used to identify the cases with re-used devices and the controls with new devices. Data were extracted from clinical notes in the cardiac clinic and additional information from pacemaker cards in the cardiac catheterisation laboratory and clinical records. Patient status was taken from clinical notes, the hospital electronic record (Clinicom) and the records of the Department of Home Affairs.

## Statistical analysis

Categorical data were summarised as proportions and continuous data as means and standard deviations or medians and interquartile range. Categorical data were compared using the chi-squared test, and continuous data using the Student’s *t*-test or Mann–Whitney test. All tests were two-sided and a *p*-value of < 0.05 was considered significant. IBM SPSS (version 19, IBM Corp, NY, USA) was used to perform the analysis.

## Results

Three patients with re-used pacemakers were excluded from the analysis because of missing records. Data for 126 devices inserted in 126 patients between 2003 and 2013 were analysed, of which 102 (81%) were pacemakers (51 re-used and 51 new) and 24 (19%) were ICDs (12 re-used and 12 new). For the pacemaker group, the median follow up for patients with re-used devices (cases) was 15.1 months [interquartile range (IQR), 1.3–36.24 months] and for those with new devices (controls) it was 55.8 months (IQR, 20.3–77.8 months). In the ICD group, the median follow up for patients with re-used devices (cases) was 35.9 months (IQR, 17.0–70.9 months) and for those with new devices (controls) it was 45.7 months (IQR, 37.6–53.7 months).

Baseline characteristics of patients who received pacemakers are shown in Table 1 and pacemaker parameters are shown in Table 2. As expected, the re-used pacemaker cases had more significant co-morbidities compared to the pacemaker controls. They were more likely to have advanced cancer, CVA, advanced COPD and dementia, with a poor baseline level of functioning, mainly bed bound (due to CVA, dementia, atherosclerotic and diabetic vasculopathies with lower limb amputations and arthritis). There were no differences between the two groups with regard to pacemaker parameters, as shown in Table 2.

**Table 1:** Characteristics of patients who received pacemakers

*Characteristics*	*Patients with re-used pacemakers (cases)*	*Patients with new pacemakers (controls)*	*p-value*
Sample size, *n*	51	51	
Age, years	74.33 ± 17.26	72.86 ± 16.13	0.658
Gender, *n* (%)			
Male	24 (47.1)	24 (47.1)	1.00
Female	27 (52.9)	27 (52.9)	
Co-morbidities, *n* (%)			
Hypertension	26 (51)	35 (68.6)	0.069
Diabetes mellitus	7 (13.7)	13 (25.5)	0.135
Renal impairment	17 (33.3)	19 (37.3)	0.679
Cancer	7 (13.7)	3 (5.9)	0.49
Myocardial infarction	6 (11.8)	11 (21.6)	0.29
Cardiomyopathy	4 (7.8)	6 (11.8)	0.74
CVA	12 (23.5)	3 (5.9)	0.02
COPD	5 (9.8)	1 (2)	0.21
Dementia	10 (19.6)	1 (2)	0.008
Baseline function, *n* (%)			
NYHA functional class 1	3 (5.9)	7 (13.7)	0.32
NYHA functional class 2	15 (29.5)	27 (52.9)	0.026
NYHA functional class 3	14 (27.5)	14 (27.5)	1.00
Wheelchair bound	4 (7.8)	3 (5.9)	1.00
Bed bound	15 (29.4)	0 (0)	< 0.0001
Indications			
Sick sinus syndrome, *n* (%)			
Yes	9 (17.6)	4 (7.8)	0.138
No	42 (82.4)	47 (92.2)	
AV block, *n* (%)			
Yes	38 (74.5)	43 (84.3)	0.22
No	13 (25.5)	8 (15.7)	
Other, *n* (%)			
Yes	4 (7.8)	4 (7.8)	
No	47 (92.2)	47 (92.2)	1.00
First implantation, *n* (%)	43 (84.3)	45 (88.2)	0.565
Battery change, *n* (%)	8 (15.7)	6 (11.8)	0.565
Primary implanter			
Cardiologist	25	25	1.00
Cardiology registrar	26	26	1.00
Temporal lead, *n* (%)	17 (33.3)	21 (41.2)	0.413
Follow up at 3 months, *n* (%)			
Yes	26 (51)	43 (84.3)	< 0.0001
No	25 (49)	8 (15.7)	
Follow up at 1 year, *n* (%)			
Yes	19 (37.3)	38 (74.5)	< 0.0001
No	32 (62.7)	13 (25.5)	

CVA = cerebrovascular accident; COPD = chronic obstructive pulmonary disease; NYHA = New York Heart Association; AV block = atrioventricular block; n = number; (%) = percentage; Other = atrial fibrillation and heart failure.

**Table 2:** Pacemaker parameters

*Parameters*	*Patients with re-used pacemakers (cases)*	*Patients with new pacemakers (controls)*	*p-value*
DDD, *n* (%)	11 (21.6)	7 (13.7)	0.30
VVI, *n* (%)	39 (76.5)	42 (82.4)	0.463
Other, *n* (%)	1 (2)	2(3.95)	
Minimum pacing rate, bpm	63.4 ± 6.0	61.6 ± 5.1	0.09
Ventricular pacing, *n* (%)	50 (98)	49 (96.1)	0.558
Battery voltage, V	2.78 (2.77–2.79)		
Battery current, A	13.86 ± 4.9		
Battery impedance, KΩ	0.482 ± 0.3		
Estimated battery life (years)	6.085 ± 1.7		
Capture			
Amplitude, V			
Atrial	0.48 ± 0.15	0.57 ± 0.23	0.323
Ventricular	0.49 ± 0.34	0.48 ± 0.18	0.747
Pulse width, ms			
Atrial	0.5 (0.5–0.5)	0.5 (0.475–0.5)	0.485
Ventricular	0.5 (0.5–0.5)	0.5 (0.5–0.5)	0.355
Sensitivity, mV			
Atrial	4.3 (3.750–5.5)	3.8 (2.875–6.2)	0.255
Ventricular	14.09 ± 6.50	15.27 ± 7.14	0.406
Electrode impedance, Ω			
Atrial	692 ± 178	804 ± 275	0.289
Ventricular	748 ± 267	808 ± 285	0.289

Other = AAI, V = volts; mV = millivolts; ms = millisecond; Ω = ohms; KΩ = kilo-ohms; A = amperes; bpm = beats per minute; DDD = dualchamber pacemaker; VVI = single-chamber pacemaker.

Baseline characteristics of patients who received ICDs are shown in Table 3 and there were no significant differences between the two groups. ICD parameters are shown in Table 4 and there were no significant differences between the two groups.

**Table 3:** Characteristics of patients who received implantable cardioverter defibrillators

*Characteristics*	*Patients with re-used ICDs (cases)*	*Patients with new ICDs (controls)*	*n-value*
Sample size, *n*	12	12	
Age	49.83 ± 17.34	50.58 ± 17.27	0.916
Gender, *n* (%)			
Male	10 (83.3)	10 (83.3)	
Female	2 (16.7)	2 (16.7)	
Co-morbidities, *n* (%)			
Hypertension	4 (33.3)	4 (33)	1.00
Diabetes mellitus	1 (8.3)	2 (16.7)	0.537
Renal impairment	8 (66.7)	6 (50)	0.408
Cancer	0 (0)	0 (0)	
Myocardial infarction	7 (58.3)	4 (33.3)	0.49
Cardiomyopathy	3 (25)	2 (1.7)	1.00
CVA	1 (8.3)	1 (8.3)	1.00
COPD	2 (1.7)	0 (0)	0.48
Dementia	0 (0)	0 (0)	
Baseline function, *n* (%)			
NYHA functional class 1	1 (8.3)	5 (41.7)	0.20
NYHA functional class 2	7 (58.3)	7 (58.3)	1.00
NYHA functional class 3	4 (33.3)	0 (0)	0.11
Wheelchair bound	0 (0)	0 (0)	
Bed bound	0 (0)	0 (0)	
Ventricular tachycardia, *n* (%)	9 (75)	10 (83.3)	0.615
Other, *n* (%)	3 (25)	2 (16.7)	0.615
First implantation, *n* (%)	12(100)	11(91.7)	0.307
Battery change, *n* (%)	0(0)	1 (8.3)	0.307
Primary implanter, *n* (%)			
Cardiologist	11 (91.7)	12 (100)	1.00
Cardiology registrar	1 (8.3)	0 (0)	
Follow up at 3 months, *n* (%)			
Yes	12 (100)	12 (100)	1.00
No	0 (0)	0 (0)	
Follow up at 1 year, *n* (%)			
Yes	12 (100)	11 (91.7)	0.307
No	0 (0)	1 (8.3)	

CVA = cerebrovascular accident; COPD = chronic obstructive pulmonary disease; NYHA = New York Heart Association; AV block = atrioventricular block; n = number; (%) = percentage; Other = ventricular fibrillation and arrhythmogenic right ventricular cardiomyopathy.

**Table 4:** Implantable cardioverter defibrillator parameters

*Parameters*	*Patients with re-used ICDs (cases)*	*Patients with new ICDs (controls)*	*p-value*
VVI, *n* (%)	12	12	1.00
Minimum pacing rate, bpm	38.1 ± 4.7	44.4 ± 9.4	0.052
Ventricular pacing, %	12	12	1.00
Capture			
Amplitude, V			
Ventricular	0.618 ± 0,28	0.708 ± 0.32	0.481
Sensitivity, mV			
Ventricular	12.925 ± 6.93	16.118 ± 6.17	0.258
Output			
Amplitude, V			
Ventricular	3.5 (3.3–3.875)	3.5 (3–3.5)	0.875
Electrode impedance, Ω			
Ventricular	784.75 ± 304	648.83 ± 147	0.177

V = volts; mV = millivolts; ms = milliseconds; Ω = ohms; KΩ = kilo-ohms; A = amperes; bpm = beats per minute; VVI = single-chamber device.

The pacemaker group was analysed separately from the ICD group. In the pacemaker group there were no device infections, pacemaker malfunction, early battery depletion or explantation of pacemaker due to infection, malfunction and early battery depletion identified after a median follow up of 15.1 months (IQR, 1.3–36.24 months) for the cases and 55.8 months (IQR, 20.3–77.8 months) for the controls.

For the pacemaker cases, 10 (19.6%) patients were followed up for five years or more, 18 (35.3%) for one to five years, and 23 (45.1%) for less than a year. For the pacemaker controls, 23 (45.1%) patients were followed up for five years or more, 21 (41.2%) for one to five years, and seven (13.7%) for less than a year.

In the ICD group, there was one device in the re-used device group that delivered inappropriate shocks (inappropriate delivery of shocks for supraventricular tachycardia), during the early stages of implantation but this resolved without any intervention. This device subsequently needed generator replacement after 14 months from implantation. There were no device infections identified after a median follow up of 35.9 months (IQR, 17.0–70.9 months) for the cases and 45.7 months (IQR, 37.6–53.7 months) for the controls. There were no procedure-non-related infections documented for the follow-up period.

For the ICD cases, five (41.7%) patients were followed up for five years or more, and seven (58.3%) for one to five years. For the ICD controls, seven (58.3%) were followed up for five years or more, and five (41.7%) for one to five years. In both groups (pacemaker and ICD) there were no devices explanted for infection or malfunctioning during the follow-up period.

**Figure 2.**
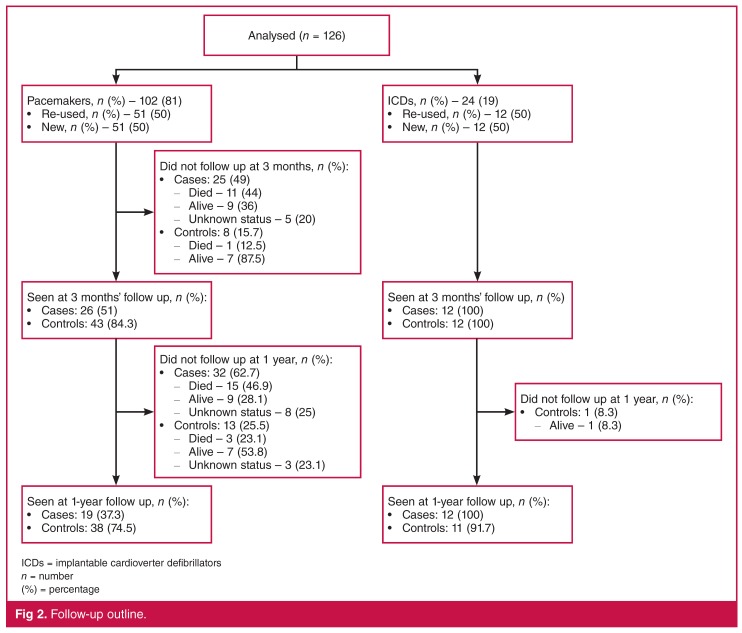
Follow-up outline.

In the re-used pacemaker group, 26 (51%) patients attended follow up at three months, whereas 25 (49%) did not attend. Of those who did not attend, 11 (44%) had died, nine (36%) were alive, and five (20%) were lost to follow up (Fig. 2). Of those who died, eight (72.7%) were documented to have died from natural causes, one (9.1%) from cancer and two (18.2%) from non-pacemaker-related sepsis, of whom one died within 24 hours of implantation and the other after two months of implantation. The patient who died within 24 hours of device implantation was admitted with a methicillin-resistant *Staphylococcus aureus* (MRSA) endocarditis prior to pacemaker implantation.

In the new pacemaker group, 43 (84.3%) patients attended follow up at three months, whereas eight (15.7%) did not attend follow up. Of those who did not attend, one (12.5%) had died and seven (87.5) were alive (Fig. 2). The patient who died was an 87-year-old man who passed away at home two days after pacemaker implantation from natural causes.

In the re-used pacemaker group, at one-year follow up, 19 (37.3%) patients attended follow up, whereas 32 (62.7%) did not attend follow up. Of those who did not attend follow up, 15 (46.9%) had died, nine (28.1%) were alive, and eight (25%) were lost to follow up (Fig. 2). All deaths were due to natural causes except the two who were septic, mentioned above.

For the new pacemaker group, 38 (74.5%) patients attended follow up while 13 (25.5%) patients did not attend follow up at one year. Of those who did not attend follow up, three (23.1%) had died, seven (53.8%) were alive and three (23.1%) were lost to follow up (Fig. 2). All deaths were due to natural causes.

In the ICD group, there was 100% attendance for both cases and controls at three months’ follow up. At the one-year follow up, there was 100% attendance for the cases compared to 91.7% for the controls, with one (8.3%) patient absent. However, this patient had been discharged from Groote Schuur Hospital at three months of follow up, to be followed in Port Elizabeth, and was still alive at the time of publication (Fig. 2).

## Discussion

This study shows that the re-use of pacemakers and ICDs was feasible and safe in our group of patients at Groote Schuur Hospital in Cape Town, South Africa. There was no difference in the incidence of device infection, malfunction, battery failure or explantation due to complications between re-used and new devices. Indeed, device implantation was associated with no complications in this series.

To the best of our knowledge this is the second study ever published of the outcomes of re-used ICDs.^24^ In our study, there were no identified device infections and/or devices explanted for malfunction. There were no patients who were lost to follow up in this group.

Linde *et al.*,^22^ in a retrospective case–control study, found no significant difference in device infection, although paradoxically, more infections were found in the new pacemaker group (7%) than in the re-used pacemaker group (2%). Kantharia *et al.*^25^ found no significant complications in an Indian study cohort of 53 patients who received cadaveric donated resterilised pacemakers over a mean follow up of 661 days.

Panja *et al.*^26^ found no difference in infection rates between the new pacemaker group and cadaver-donated re-used pacemakers. However, higher rates of infection were found on infected re-sterilised devices that were implanted in the same patient, which were taken out and implanted on the opposite side. They attributed this higher infection rate to haematogenous or lymphatic spread from the previously infected pocket.^26^ Rosengarten *et al.*^27^ also found no significant difference in major pacemaker-related complications and reported that re-use of devices is cost effective.

Pavri *et al.*,^24^ in a retrospective, single-centre cohort study of re-sterilised ICDs found no device-related infections, and 60.4% re-used ICDs delivered life-saving shocks. Baman *et al.*,^28^ in a meta-analysis of 18 studies, found no significant difference in infection rates between the new device group and the re-used device group, but much higher device malfunction was associated with re-used devices compared to new devices. This malfunction was attributed to abnormality in the set screws.^28^

In a recent study, Nava *et al.*^23^ found no significant difference in infection rates between re-used and new devices, although more infections were found in the new device group. They also found more device malfunction in the re-use device group, which was similar to the above studies, and the fault was also attributed to faulty pacemaker screws.^23^

Device infection is thought to be associated with mortality rates between 2.6 and 18%.^12^-^14^ However studies that examined this issue showed no significant difference in infection or mortality rates between re-used and new device implantation.^14^-^22^ In our study we did not compare mortality rates between the two groups because of the selection bias of those who received a re-used pacemaker.

From the findings of this study and also acknowledging its limitations, pacemaker and ICD re-use is feasible and safe. It is a reasonable option for those who cannot afford new devices, provided that proper selection and sterilisation measures of re-used devices are followed. In the developing world, where there are major resource constraints, this option should be explored for the benefit of those suffering from symptomatic bradyarrhythmias and life-threatening tachyarrhythmias.

We acknowledge several limitations of our study. First, this was a retrospective study with a small sample size of cases with re-used pacemakers and ICDs. Second, the follow-up period of patients with re-used devices was relatively short, with a median period of 15 months, with a significant number of patients who died within three months of device insertion. Finally, the patients who were selected for re-used pacemakers had significant co-morbidities, which were associated with a shortened life-span. These factors may limit the generalisability of the study, and call for appropriate prospective studies to answer this question.

## Conclusion

Pacemaker and ICD re-use is feasible and safe in the short term (i.e. over months) provided that the devices for re-use are selected carefully and proper sterilisation methods are followed. Re-used pacemakers and ICDs are a realistic option for patients with co-morbidities who live in developing countries where there is limited access to pacemakers and ICDs.
